# Preventive effect of methenamine in women with recurrent urinary tract infections – a case–control study

**DOI:** 10.1080/02813432.2022.2139363

**Published:** 2022-11-11

**Authors:** Linda Rui, Morten Lindbaek, Svein Gjelstad

**Affiliations:** Department of General Practice, Antibiotic Centre for Primary Care, Institute for Health and Society, University of Oslo, Oslo, Norway

**Keywords:** Recurrent infection, urinary tract infection, methenamine, antibiotics, women

## Abstract

**Background:**

Urinary tract infection (UTI) is the most common bacterial infection in women. In Norwegian general practice, methenamine has been prescribed for many years as long-term prevention and accounted for 20% of the total antibiotic prescribing in 2015, as measured in defined daily dosages (DDDs). The efficacy of methenamine is unknown. If shown to be effective, this drug may become an important preventive against UTI.

**Objective:**

To examine whether methenamine is preventive against recurrent UTI in women.

**Design:**

Data for all antibiotics used for UTIs dispensed from all pharmacies from 2005 to 2015 were collected from the Norwegian prescription database (NorPD).

**Subjects:**

Women aged ≥ 40 years with recurrent UTI, defined as ≥3 courses of UTI antibiotic/year, were included.

**Main outcome measures:**

Patients using methenamine (cases) and those not using methenamine (controls) were compared. The numbers of UTI prescriptions during the 2 years before and after inclusion were analysed. **Results:** The yearly prevalence for recurrent UTI was 2.4% in women ≥ 40 years. The change in antibiotic use from 2 years before to 2 years after inclusion in the study differed significantly between groups: 44.6 and 34.9% reductions in the number of antibiotic prescriptions for UTI in the methenamine and control groups, respectively. The decrease in UTI antibiotic prescriptions (58.9%) was greater in patients with a higher consumption of antibiotics before starting methenamine.

**Conclusions:**

Methenamine seems to be effective against recurrent UTI over the time span studied. The effect seems to be greater in patients with the highest number of recurrent UTIs.

**Key points**
- Methenamine has been used for many years for prevention of recurrent UTI, but no studies have demonstrated a significant preventive effect of long time use.
- This study shows that methenamine seems to be effective for prevention in patients having recurrent UTI over 2 years or more.
- The effect seems to be larger in patients with a high number of UTIs over 2 years.

## Background/introduction

Urinary tract infection (UTI) is the most common condition that requires antibiotic treatment in women, and treating UTI recurrence is a common challenge [1–9]. *Escherichia coli* is the most frequent microbe causing uncomplicated UTI [10–13], and antimicrobial resistance (AMR) is an increasing global problem. Enterobacterales, which cause most UTIs, have shown increasing resistance against the antibiotics used most often to treat UTIs. In Norway, up to 30% of women aged ≥ 40 years report symptoms of recurrent UTI [14]. However, few studies have analysed antibiotic use for recurrent UTI, and an important issue relates to the feasible and appropriate preventive measures to prevent and treat recurrent UTI.

Methenamine is often prescribed in Norway and Sweden as a preventive against recurrent UTI [8,9,15]. Methenamine hippurate exerts antibacterial activity through the conversion of methenamine to formaldehyde in the presence of acidic urine. Methenamine hippurate is generally active against *E. coli* and *Enterococcus* and *Staphylococcus* species. However, inhibition of *Proteus* and *Pseudomonas* is achieved only in acidic urine, and optimization of the pH of the urine is recommended. Methenamine acts as a bacteriostatic agent, and no antibiotic resistance has been reported.

A 2012 Cochrane review on methenamine examined the effects and advantages of methenamine for the prevention of UTIs [16]. Thirteen studies with 2032 participants met the inclusion criteria. Six trials with a total of 654 patients reported on symptomatic UTIs, eight trials involving 796 patients reported the effects on bacteriuria, and one reported both outcomes. No studies were found on the longstanding use of methenamine for the prevention of recurrent UTI. In that review, subgroup analyses indicated that methenamine may have efficacy in patients without urinary or kidney abnormalities. For example, for the short-term treatment [≤1 week], a reduction in the number of symptomatic UTIs was found in patients without urinary or kidney abnormalities. The authors concluded that methenamine may be effective for the prevention of UTIs when used as a short-term treatment. The rates of adverse events for preventing UTIs are low [15], but more studies are needed to assess both the safety and efficacy of the preventive use of methenamine hippurate over the longer term [17–22].

Although Norway has a lower rate of antibiotic consumption than many other countries, there is potential for improvement [15,23]. The national guidelines recommend more restrictive prescribing practices, especially for respiratory tract infections (RTIs) and UTIs [23]. The typical antibiotics for UTIs (pivmecillinam, trimethoprim, co-trimoxazole, nitrofurantoin and ciprofloxacin) constituted 22% and methenamine 20% of the total number of antibiotic prescriptions, as measured in DDDs [24].

A 2015 review concluded that antibiotics are still the most effective preventive treatment for recurrent UTI, although documentation of the effects of methenamine is still lacking [3]. A 2016 review evaluated the evidence for the effects of methenamine and other antimicrobials for UTIs and the development of AMR. That review concluded that it is essential to limit the use of resistance-driving antibiotics and to generate new knowledge about older preventive agents, such as methenamine, to limit antibiotic resistance [25]. A recent study from urological departments in UK found that methenamine was non-inferior to prophylactic antibiotics in patients with recurrent UTIs [26]. To our knowledge, no adequately powered studies on the long-term effects of methenamine in primary care have been reported. If methenamine proves to be effective, it may provide a means for reducing the prescription of resistance-driving antibiotics to treat UTIs.

The purposes of this study were to:

- analyse the frequency of antibiotic use for recurrent UTI in Norway in women aged ≥ 40 years,
- describe the use of methenamine in Norwegian women,
- assess the effects of methenamine in the prevention of recurrent UTI in women aged ≥ 40 years.

Recurrent UTI was defined as the prescription of ≥3 antibiotic courses within 12 months. We compared the number of infections in women prescribed methenamine for recurrent UTI (cases) with that in women with recurrent UTI who had not been prescribed methenamine (controls).

## Materials and methods

All dispensations of the common antibiotics used to treat UTIs retrieved from the Norwegian prescription database (NorPD) in the 10-year period July 2005 to June 2015 were extracted. These included the antibiotics methenamine, pivmecillinam, nitrofurantoin, trimethoprim, co-trimoxazole and ciprofloxacin. Given that NorPD has no diagnostic codes for antibiotic dispensations from pharmacies, there was some uncertainty about the reasons for the dispensations of the various prescriptions However, a recent article, giving national prescriptions for UTI in consultations in the time period *2006–2015* [27] found that ciprofloxacin comprised 7.3% of all antibiotics given for UTI (the five UTI-antibiotics pivmecillinam, trimethoprim, nitrofurantoin, ciprofloxacin and cotrimoxazole). The corresponding number for cotrimoxazole was 6.1%. In addition, we have found the corresponding proportion of total national antibiotic prescriptions in the same period, comprising all diagnoses (NOrPD) and ciprofloxacin comprised 13.6% and cotrimoxazole comprised 8.8%. By comparing the numbers, we can conclude that UTI-prescriptions of ciprofloxacin comprised 54% of all prescriptions, and the corresponding proportion for cotrimoxazole was 70%. Amoxicillin was excluded because it is used more often for infections other than in the urinary tract, mainly for acute RTIs.

Figure 1 shows the flow chart of the study participants, starting with all women who received an antibiotic prescription in the 10-year period from 1 July 2005 to 30 June 2015. The inclusion criteria were ≥3 dispensations for UTIs in a 12-month period within the 2-year observation time, age ≥ 40 years, 2 years of observation data before and after the time frame of 1 July 2007 to 30 June 2013, and regular minimum use of methenamine over a given time period, defined as ≥0.5 DDD/d per 2 years. We excluded patients receiving dispensations of >50 doses of antibiotics used to treat UTI because such a high number could hide recurrent UTI episodes or could indicate the use of these drugs for prevention. We excluded women aged < 40 years for practical reasons, while the risk of having recurrent UTIs increases with higher age. We chose to extend the time period to 2 years to ensure that we had 2 years of observations without methenamine before and after inclusion. Another reason for the choice of 2 years for observation of those with 3–5 UTI prescriptions was that preliminary studies have shown large variations with only 1 year of observation, and we wanted to include patients with a stable high prescription rate.

Figure 1.Flow chart for the inclusion and exclusion of patients, data obtained from NorPD.

**Figure:**
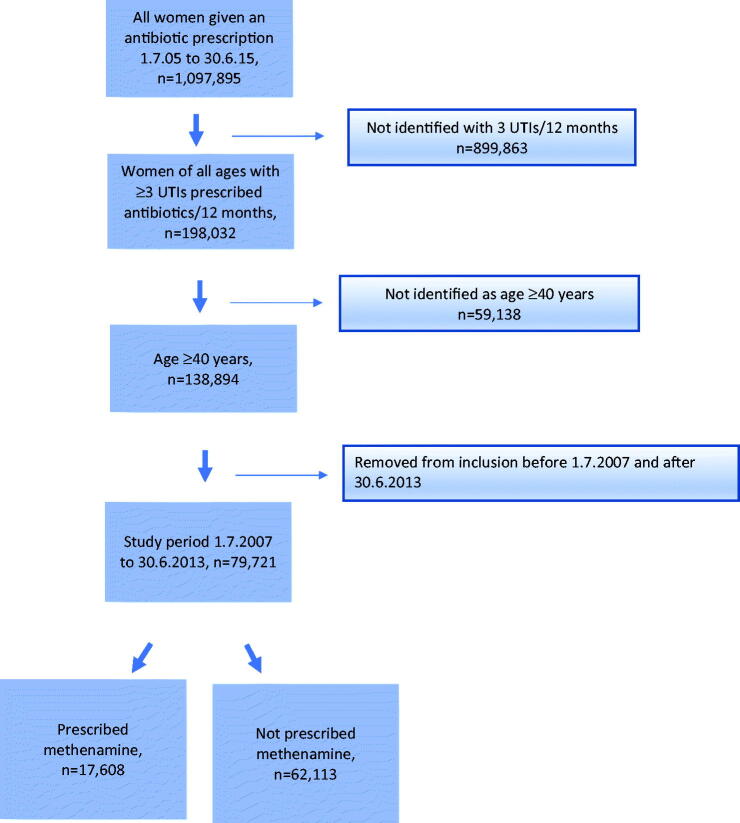

**Figure:**
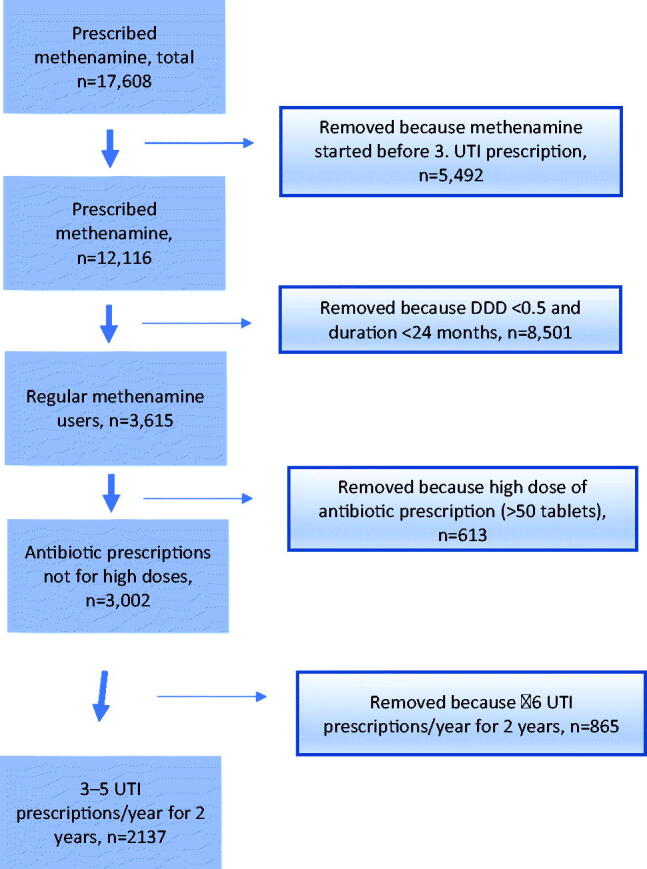

The control group was matched for age with the methenamine-treated group (cases) within a span of 10 years, year of inclusion, and the number of antibiotic prescriptions per 2-year period. Those included in the control group had not been prescribed methenamine in the study period.

The starting point for analysis of the methenamine group was when the patient started regular use of methenamine. We compared the number of prescriptions for UTI for the 2 years before and after this point. The inclusion period was defined as the start of treatment with ≥3 prescriptions of methenamine per year and methenamine consumption of ≥0.5 DDD/d in the methenamine group, and ≥3 UTIs/year in the control group. In the main material, we linked patients who had 3–5 prescriptions/year within a 2-year period, which produced 2137 patients in each of the groups.

We looked specifically at the group of patients who used methenamine and who received >5 prescriptions for UTI/year within a 2-year period. These were not compared with a control group because there was an insufficient number of patient to make a meaningful comparison. We analysed only the number of prescriptions for UTI before and after the start of methenamine in this group of high consumers.

For statistical analysis, we used the *t* test to compare groups, using IBM SPSS statistics (IBM Corp., Armonk, NY).

## Results

**Figure 2.**
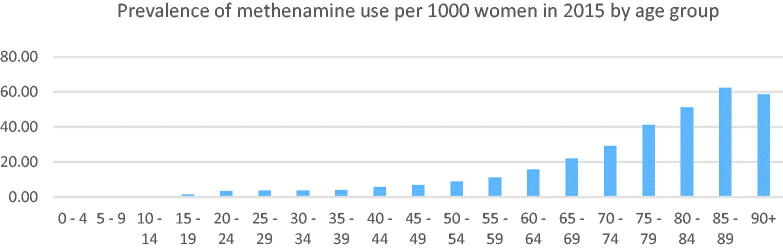
Prevalence of methenamine use per 1000 women in 2015 in Norway, by age group, data obtained from NorPD [28].

The prevalence rates per 1000 women of methenamine prescriptions per 5-year age groups in 2015 are shown in Figure 2. The prevalence increased from age 50 years, and the highest prevalence of 60/1000 women was in the 85–90-year group.

Our search of the NorPD showed that the total number of patients receiving antibiotics in women aged 60–80 years increased from 120,000 to 150,000 in the period 2004–2014, which is consistent with the relative increase in size of this age group within the population. During the same period, the number of methenamine users increased from 5000 to 10,000 [28].

In the 10-year period 2006–2015, we found that 200,000 women had received ≥3 prescriptions for UTI (Figure 1) and that 12% of women aged ≥ 40 years had received a prescription for UTI in 2016. Using the limit of a minimum of 20 DDDs to define high use (e.g. three courses with 7 DDDs/course), we classified 19% of the women aged ≥ 40 years getting UTI antibiotics, as high users of antibiotics for UTIs, which equated to 2.4% of all women in this age group. Table 1 shows the use of the total prescriptions per 1000 women for the 5 UTI-antibiotics in the time period given. There was a trend of increasing number of UTI prescriptions over the years until 2014, which is also confirmed in the paper by Haugom et al. [27]. The proportion of mecillinam has risen in the period, while Trimethoprim has fallen, the other 3 have been stable over time.

**Table 1.** National data of prescriptions of antibiotics for urinary tract infections per 1000 women (%) per year in 2006–2015 (source NorPD).

Antibiotic	2006	2007	2008	2009	2010	2011	2012	2013	2014	2015
Pivmecillinam	68 (48)	74 (50)	80 (52)	83 (54)	87 (55)	87 (55)	88 (56)	91 (57)	92 (58)	89 (58)
Trimethoprim	44 (31)	42 (29)	40 (26)	37 (24)	36 (23)	34 (22)	32 (20)	31 (19)	30 (19)	27 (18)
Nitrofurantoin	11 (8)	11 (7)	11 (7)	12 (8)	13 (8)	14 (8)	14 (9)	14 (9)	14 (9)	14 (9)
Sulfamethoxazole	9 (7)	9 (6)	9 (6)	9 (6)	9 (6)	9 (6)	9 (6)	10 (6)	12 (7)	12 (8)
Ciprofloxacin	10 (7)	12 (8)	13 (8)	13 (8)	14 (8)	14 (8)	15 (9)	14 (9)	13 (8)	11 (7)
Sum	143 (100)	147 (100)	154 (100)	154 (100)	159 (100)	159 (100)	158 (100)	160 (100)	160 (100)	154 (100)

We found in total 17,608 patients using methenamine in the 6-year period, and we included 3002 patients in the material. Table 2 shows the difference in dispensations for the 2 years before and after the start of methenamine and a comparison with the control group of women with recurrent UTI who did not use methenamine in the same time period. The average number of dispensations decreased by 1.75 in the methenamine group and by 1.16 in the control group, which was equivalent to reductions of 45% and 35%, respectively, in the mean number of dispensations per year (*p* < 0.001). The absolute risk reduction was 45–35%=10%, giving numbers needed to treat (NNT) of 100/10 = 10, meaning that 10 patients should be treated with methenamine to avoid one episode of UTI. The mean number of prescriptions was slightly higher in the first period in the methenamine group than in the control group (3.95 *vs.* 3.35).

**Table 2.** Differences in the dispensation of antibiotics for the treatment of UTIs in women aged ≥ 40 years using methenamine >0.5 DDD/d for 2 years compared with a matched control group with ≥ 3 UTIs/12 months before inclusion.

	Methenamine users *n* = 2137 patients		Control group *n* = 2137 patients	
	2 years before inclusion	2 years during the methenamine treatment period	2 years before inclusion	2 years after inclusion
Mean number of antibiotic dispensations from the pharmacy (95% confidence interval)	3.90 (3.86–3.94)	2.15 (2.06–2.24)	3.35 (3.33–3.38)	2.19 (2.09–2.28)
Mean change after *vs.* before inclusion		–1.75 (–1.85 to −1.65)		–1.16 (–1.26 to −1.07)
Change in percent		–44.9 (–47.9 to −41.9)		–34.6 (–37.8 to −31.7)

Data from the NorPD.

The analysis of the number of antibiotic prescriptions in the high consumers of antibiotics in the corresponding 2-year period, defined as ≥6 prescriptions/year, is shown in Table 3. The high consumers of antibiotics had a large absolute reduction in the number of prescriptions, and the relative reduction was 58.1%. However, we had no control group for these patients. The decrease in the number of prescriptions was significantly higher than the 35% decrease in the control group that had a moderate consumption of antibiotics.

**Table 3.** Differences in the dispensation of antibiotics for UTI in women aged ≥ 40 years using methenamine > 0.5 DDD/d for 2 years with a high number of dispensations per year (>5/year), data obtained from NorPD.

	Methenamine users *n* = 865 patients		Control group No matching patients	
	2 years before inclusion	2 years during the methenamine treatment period	2 years before inclusion	2 years after inclusion
Mean number of antibiotic prescriptions from the pharmacy (95% confidence interval)	7.06 (6.97–7.15)	2.95 (2.79–3.12)	N/A	N/A
Mean change after *vs.* before inclusion		–4.10 (–4.28 to −3.93)	–	–
Change in percent		–58.1 (–61.4 to −55.0)	–	–

Since ciprofloxacin is also prescribed for other infections such as RTIs, we performed a sensitivity analysis where ciprofloxacin was excluded from the analysis. We found that the reduction of UTI prescriptions was 1.93 (95% CI 1.88–2.02) or 49% in the methenamine group *vs.* 1.66 (95% CI 1.56–1.76) or 42% in the control group (*p* < 0.05). This gives a difference of 7% between the two groups. In the high prescription group the reduction was 4.26 prescriptions or 61%.

## Discussion

### Main findings

The main result of this study is that methenamine seems to have a preventive effect in women over 40 years with recurrent UTI, defined as ≥3 prescriptions for UTI per year over 2 years, compared with the control group. The 10% difference between the groups is regarded as clinically significant. The women with more frequent UTI prescriptions seem to have exhibited a larger effect of methenamine based on the larger reduction in the number of UTI prescriptions in patients with 6 UTIs/year than in those with 3–5 UTIs/year. However, we cannot conclude definitely given that it was impossible to find a valid control group in our material, mainly because almost all of these patients received methenamine.

Another important observation is the spontaneous variation in the patients who did not receive methenamine. These patients exhibited a 35% reduction in the number of prescriptions from the first 2 years to the next period. This finding suggests that, in some women, the frequency of recurrent UTI may be high in any given year but may be much lower in the following years. This is important for GPs considering when to start preventive treatment with methenamine.

Our data also show that recurrent UTI is a frequent diagnosis among Norwegian women aged ≥40 years and is associated with the prescription of many antibiotic courses, which may contribute to the development of AMR. Methenamine may be a valuable tool for trying to reduce the number of antibiotic prescriptions, although well-powered RCTs are necessary to confirm this. The number of methenamine users among women aged 60–80 years doubled from 2005 to 2015, whereas the number of women with UTI-prescribed antibiotics increased by 25%, in line with the increase in the proportion within this age group in the general population [28,29]. In other countries, long-term treatment with antibiotics such as trimethoprim or nitrofurantoin is used for the prevention of recurrent UTI, but this may increase the risk for AMR. Methenamine has not been proven to cause AMR and may be a better alternative [15].

We have compared the use of the 5 UTI-antibiotics for women in Norway and Sweden. In Sweden, there was a significant increase of mecillinam and nitrofurantoin, and a corresponding decrease of trimethoprim/sulfamethoxazole in the years 2006–2015 [30]. This is possibly due to that trimethoprim is no longer recommended as first line treatment for UTI. In 2015, the proportion of women treated with UTI was similar in the two countries. Mecillinam and nitrofurantoin were the most commonly used UTI-antibiotics, comprising 76% of the total in Sweden, while mecillinam and trimethoprim comprised 75% of the total in Norway in 2015.

### Strengths and weaknesses

The strength of this large study is that it was based on all prescriptions, for both antibiotics and methenamine, in Norway in the included 6 years, which means there is little danger of selection bias. We assume that the data analysed are representative of clinical practice in the time span studied in Norway. We used strict inclusion criteria to ensure sufficient observation periods before and after inclusion and the regular use of methenamine. This meant that only 17% of all methenamine users were included in the study, which suggests that our results are representative only of regular users.

The prescription rate was higher in the methenamine group than in the control group. Assuming a regression to the mean effect, this may have led to the larger reduction in the rate in the methenamine group.

The NorPD data are based on prescriptions and dispensations and we cannot be sure that the patients actually took the drugs. There may have also been a source of error related to the diagnosis of UTI because we did not know whether the patients who received these prescriptions actually had UTI. There are guidelines for the diagnosis of UTI, but we do not know how often these were followed in clinical practice.

We also have no data about the pH of the urine of the patients who received methenamine. Although most people initially have acidic pH, some have basic urine. We assume that these patients would have little effect of methenamine, thus giving a smaller difference between the groups.

Most of the antibiotics included in this study are used to treat UTIs and rarely for other diagnoses. However, although ciprofloxacin is used mostly for UTIs, it has other indications, such as RTIs. We assumed that ciprofloxacin was prescribed in the same manner in both groups and that this provided only a small risk of bias. We performed a sensitivity analysis where ciprofloxacin was excluded and this still showed a significant difference of 7% between the two groups.

It has taken us some years to finish the analyses and write this article. Despite this delay, we think our results are valid because the prescription rates of antibiotics and methenamine for UTIs have remained stable over the past years.

### Comparison with other studies

Few studies have evaluated the preventive use of methenamine for recurrent UTI and have produced varying results [16–22,26]. Only short-term use has been shown to be effective. Our study is one of the first to provide data on the effects of long-term preventive use of methenamine in primary care. Some new RCTs have recently started to evaluate the long-term effects of methenamine [26, personal communication Cees Hertogh, leader of ImpresU].

### Implications for research and/or practice

Our findings suggest that methenamine has a significant preventive effect in women aged 40 and above with a history of recurrent UTI for ≥2 years. Methenamine has few side effects, and so far, no resistance has been reported. RCTs are needed to confirm this preventive effect.
